# Augmentation index is associated with coronary revascularization in patients with high Framingham risk scores: a hospital-based observational study

**DOI:** 10.1186/s12872-015-0123-0

**Published:** 2015-10-19

**Authors:** JoonHyouk Choi, Song-Yi Kim, Seung-Jae Joo, Ki-Seok Kim

**Affiliations:** Division of Cardiology, Department of Internal Medicine, School of Medicine, Jeju National University, #15, Aran 13gil, Jeju, 690-797 Korea

**Keywords:** Atherosclerosis, Coronary artery disease, Risk factors, Waves

## Abstract

**Background:**

This study analyzed PWAs in patients with high Framingham risk scores to determine whether PWA is predictive of coronary artery disease (CAD) severity and percutaneous coronary intervention (PCI) treatment.

**Methods:**

In total, 310 patients were screened due to suspected CAD; 78 were excluded due to PCI history (32), atrial fibrillation (11), or acute myocardial infarction (35). The augmentation index (AIx) was analyzed immediately before coronary angiography. PCI was performed in 73 (31.5 %) patients.

**Results:**

The mean AIx, adjusted by heart rate (AIx@75) was different for each clinical diagnosis in the PCI group (stable angina, 30.6 ± 7.7 %; silent ischemia, 30.2 ± 8.6 %; unstable angina, 38.5 ± 8.5 %; p = 0.026). The 10-year estimate of CVD risk, based on the Framingham heart score, was 25.3 ± 6.5 % and the mean AIx@75 was 31.6 ± 8.5 % in the PCI group, significantly higher than in the non-PCI group (18.8 ± 10.2 %, p < 0.001; 27.2 ± 9.0 %, p = 0.006, respectively). An inverse correlation was observed between the minimal luminal area and AIx@75 (rho = −0.559, p = 0.010, n = 20). In ROC curve analysis of multivariate logistic regression model, higher HDL, medication of hypertension, and higher body mass index were associated with non-PCI and higher AIx@75 was associated with PCI (area under the curve, 0.764; 95 % CI: 0.701 to 0.819, z = 8.005; p <0.001).

**Conclusions:**

The AIx@75 seemed to reflect the clinical severity of CAD and was associated with PCI in patients with a high Framingham risk score.

## Background

In the Framingham Heart Study, pulse pressure (PP) was reported to be a more important factor in cardiovascular disease (CVD) than either systolic (SBP) or diastolic blood pressure (DBP) [1]. PP, which is the difference between the SBP and DBP, has been linked with an increased prevalence of CVD and associated mortality [2–4]. An increased PP results from an increase in SBP, due to increased arterial stiffness and reflection pressure, and a decreased DBP, due to reduced arterial elasticity. Together, these factors are the elements of pulse wave analysis (PWA). The central pulse wave is composed of an early incident wave caused by left ventricular ejection and a reflected wave from the periphery [5]. As arterial stiffness increases, the velocity of the early incident wave and late reflected wave increases. If the reflected wave arrives at the central aorta early, the central aortic SBP rises. This increased pressure is called augment pressure (AP), and its percentage of the PP is called the augmentation index (AIx). The AIx@75 is adjusted to the AIx based on the heart rate [5].

Many studies have analyzed the ability of the AIx to predict coronary artery disease (CAD) severity using PWA [6–9]. In these studies, the definition of a CAD was a minimal lumen diameter (MLD) stenosis >50 %, as determined using coronary angiography (CAG). Weber *et al.* [8] showed that patients with multi-vessel CAD had high AIx values. However, PWA has not been implicated as a predictor of the need for percutaneous coronary intervention (PCI) or the patient’s clinical diagnosis. Additionally, the Framingham risk score may overestimate the CAD risk in the Chinese population outside the United States [10]. However, the reason is not clear.

The hypothesis of this study is as follows: the Framingham risk score may overestimate the CAD risk in the Korean population, because Koreans and Chinese live in the Far East Asia. If not, PWA is possible to have a lower power to predict the CAD in the patients with high Framingham risk scores. Generally, CAG was performed in patients with high Framingham risk scores and chest pain. Therefore, this research is to find out whether PWA independently can predict the clinical severity of the CAD or the need for percutaneous coronary intervention (PCI) in Korean patients with high Framingham risk scores.

## Methods

### Patient enrollment

This hospital-based observational study was approved by the ethical committee of the institutional review board of Jeju National University Hospital, and the need for written informed consent was waived due to the non-interventional nature of the study.

Between July and August 2012, 310 consecutive patients, from rural areas of Korea, were referred for elective CAG due to suspected CAD. Of these, 78 patients were excluded due to a PCI history (32), atrial fibrillation (11), or acute myocardial infarction (35). Thus, 232 patients were ultimately enrolled in the study; none had significant valvular heart diseases.

The CVD risk score was calculated using data from the Framingham Heart Study [11]. The CVD risk points included sex, age, hypertension, diabetes, smoking, total cholesterol, high density- lipoprotein (HDL), and brachial SBP. Hypertension and diabetes were defined as being present if the patients were undergoing permanent drug treatment. Current smoking status was defined as the patient having smoked at least 1 cigarette within the month prior to undergoing CAG.

Stable angina was defined as the presence of exertional chest pain, a positive stress test, and a CAG-determined MLD >50 % stenosis. Unstable angina was defined as chest pain and electrocardiographic (ECG) ST-segment depression or prominent T-wave inversion, and/or positive biomarkers of necrosis in the absence of ST-segment elevation [12], and CAG-determined MLD >50 % stenosis. Silent myocardial ischemia was defined as the absence of chest pain and CAG-determined MLD >50 % stenosis. After dividing the patients into the PCI and the non-PCI groups, we analyzed their PWAs, degrees of coronary stenosis, CAD severities, and CVD risk points.

### Coronary angiography

A consent for CAG was written. All patients underwent CAG while fasting, after having provided informed consent. Normal saline was intravenously administered before CAG to provide renal protection and to prepare for any potential emergency. Quantitative coronary angiographic analysis was performed using standard techniques and automated edge-detection algorithms (ANCOR V2.0, Siemens, Munich, Germany). Virtual histology intravascular ultrasound (VH-IVUS, Volcano Therapeutics, Rancho Cordova, CA, USA) imaging was performed after intracoronary administration of nitroglycerin (0.2 mg) using a motorized transducer pullback (0.5 mm/s). The IVUS catheter was advanced 10 mm distal to the target lesion and imaging was performed as the catheter was retracted to the aorto-ostial junction. Off-line quantitative IVUS analyses were performed with computed planimetry (Echo Plaque 3.0, Indec Systems, Mountain View, CA, USA). If diagnostic CAG failed to reveal significant stenosis (>50 % diameter stenosis, based on quantitative angiographic analysis), increasing doses of intravenous ergonovine (100 and 200 μg) were administered every 2 min until coronary artery spasms were provoked; a standard, 12-lead electrocardiogram was monitored. Variant angina was defined as chest pain and electrocardiographic evidence of myocardial ischemia and/or severe transient coronary stenosis. The PCI indications were ≥75 % stenosis, based on quantitative coronary angiographic analysis, or a minimal lumen area (MLA) <4.0 mm^2^, determined using IVUS.

### PWA

All antihypertensive medicines were withdrawn, and the patients fasted for at least 12 h prior to PWA. Brachial BP measurements and PWA were performed in the supine position after 10 min of rest, immediately prior to CAG. Brachial BP was measured before PWA by digital sphygmomanometer (Omron® M6, Omron Corporation, Kyoto, Japan). We analyzed the waveforms of each patient’s central aortic pressure using SphygmoCor® (AtCor, Sydney, NSW, Australia) after applanation of the left radial artery and recording the central aortic pressure estimate. PWA using SphygmoCor® provided central systolic, diastolic, mean, and central pulse pressures, as well as AP, AIx, and AIx@75.

### Statistical analyses

Statistical analyses were performed using SPSS 17.0 software (SPSS, Chicago, IL, USA). Data are presented as means ± standard deviations for continuous variables or as frequencies for categorical variables. Comparisons of categorical variables were performed using chi-square tests, and between-group (clinical diagnoses, the severity of CAD) comparisons were performed using one-way analysis of variance, followed by a post hoc analysis and Bonferroni correction. Spearman and Pearson correlation analysis were used to evaluate the trends between the AIx@75 and other parameters. Analysis of covariance was also run to compare the AIx@75 s between the PCI and non-PCI groups (adjusting for echocardiographically determined ejection fraction) and among clinical diagnoses (adjusting for age, HDL cholesterol level, and ejection fraction). A multiple linear regression analysis was performed to examine the predictors of AIx@75. A multivariate logistic regression analysis was performed to identify the variables independently related with the predictors of AIx@75 and to determine the probability of PCI. Receiver operating characteristics (ROC) curve analysis was used to identify the probability of AIx@75 and the risk factors, using logistic regression analysis with a forward (conditional) selection, best predicting PCI. The adequacy of the multivariable logistic regression analysis was assessed using coefficient of determination (*R*^2^) and area under curve (AUC) determinations; *p* ≤ 0.05 was considered significant.

## Results

### Baseline characteristics

In total, 73 patients (31.5 %) undergoing PCIs participated in this study. 216 patients had a mean CVD score, according to the Framingham heart study, of 16.1 ± 5.3. The 10-year estimate of their CVD risk was 20.9 ± 9.6 %. Because there were no HDL data of 16 patients, the CVD score and the 10-year estimate of CVD risk could not be calculated. In the PCI group, the clinical diagnoses included stable angina (*n* = 47), silent ischemia (*n* = 13), and unstable angina (*n* = 13). The CAD severity was expressed as 1-vessel disease (VD), 2-VD, and 3-VD. Among the patients undergoing PCI, 26 had 1-VD, 19 had 2-VD, and 28 had 3-VD. In the PCI group, the mean age was 64.8 ± 10.3 years, and the mean Framingham Heart Study CVD score was 18.6 ± 4.0 (*n* = 70). These values were significantly higher in the PCI group than in the non-PCI group (age, 59.6 ± 13.1 years; Framingham score, 14.9 ± 5.4, *n* = 146). Hypertension (72.6 % vs. 49.1 %) and hyperlipidemia (46.6 % vs. 28.9 %) were more frequently noted in the PCI group than in the non-PCI group. The 10-year CVD risk estimate was also higher in the PCI group (25.3 ± 6.5 %, *n* = 70) than in the non-PCI group (18.8 ± 10.2 %, *n* = 146; Table 1).

**Table 1:** Baseline characteristics and pulse wave analysis

	PCI group	Non-PCI group	p
	*n* = 73 (%)	*n* = 159 (%)	
Male	50 (68.5)	97 (61.0)	0.306
Age (years)	64.8 ± 10.3	59.6 ± 13.1	0.001
Height (cm)	163.8 ± 7.7	163.0 ± 8.5	0.486
Body weight (Kg)	66.9 ± 11.4	68.2 ± 11.4	0.442
Body mass index (kg/m^2^)	24.8 ± 3.5	25.6 ± 3.2	0.085
Brachial systolic pressure (mmHg)	148.9 ± 22.4	144.1 ± 22.6	0.135
Brachial diastolic pressure (mmHg)	87.0 ± 11.0	85.1 ± 10.5	0.203
Brachial pulse pressure (mmHg)	61.8 ± 17.6	58.7 ± 17.6	0.216
CVD risk points	18.6 ± 4.0	14.9 ± 5.4	<0.001*
10-year estimate of CVD risk (%)	25.3 ± 6.5	18.8 ± 10.2	<0.001*
Diabetes	20 (27.4)	33 (20.8)	0.312
Hypertension	53 (72.6)	78 (49.1)	0.001
Hyperlipidemia	34 (46.6)	46 (28.9)	0.011
Total cholesterol (mg/dL)	181.1 ± 45.6	185.9 ± 54.0	0.513
HDL cholesterol (mg/dL)	41.0 ± 11.2	46.0 ± 12.3	0.002*
Smoking	24 (32.9)	40 (25.2)	0.268
Left ventricular ejection fraction (n = 111)	59.0 ± 14.5	65.3 ± 10.1	0.036
Central pulse pressure (mmHg)	53.4 ± 17.1	49.4 ± 18.0	0.110
Augmented pressure (mmHg)	20.3 ± 9.5	16.9 ± 8.8	0.009
Augmentation index (%)	36.5 ± 9.3	32.5 ± 10.0	0.004
Augmentation index @75 (%)	31.6 ± 8.5	27.2 ± 9.0	0.006**

*CVD* cardiovascular disease, *HDL* high-density lipoprotein, *PCI* percutaneous coronary intervention, Data are expressed as means ± standard deviation or numbers (percentage); chi-square test or Wilcoxon two-sample test (or Mann–Whitney *U* test)

*p value for comparison between the PCI (*n* = 70) and non-PCI (*n* = 146); *p* < 0.05 considered significant

**ANCOVA test: adjusting for ejection fraction (*n* = 111)

### PWA

**Fig. 1:**
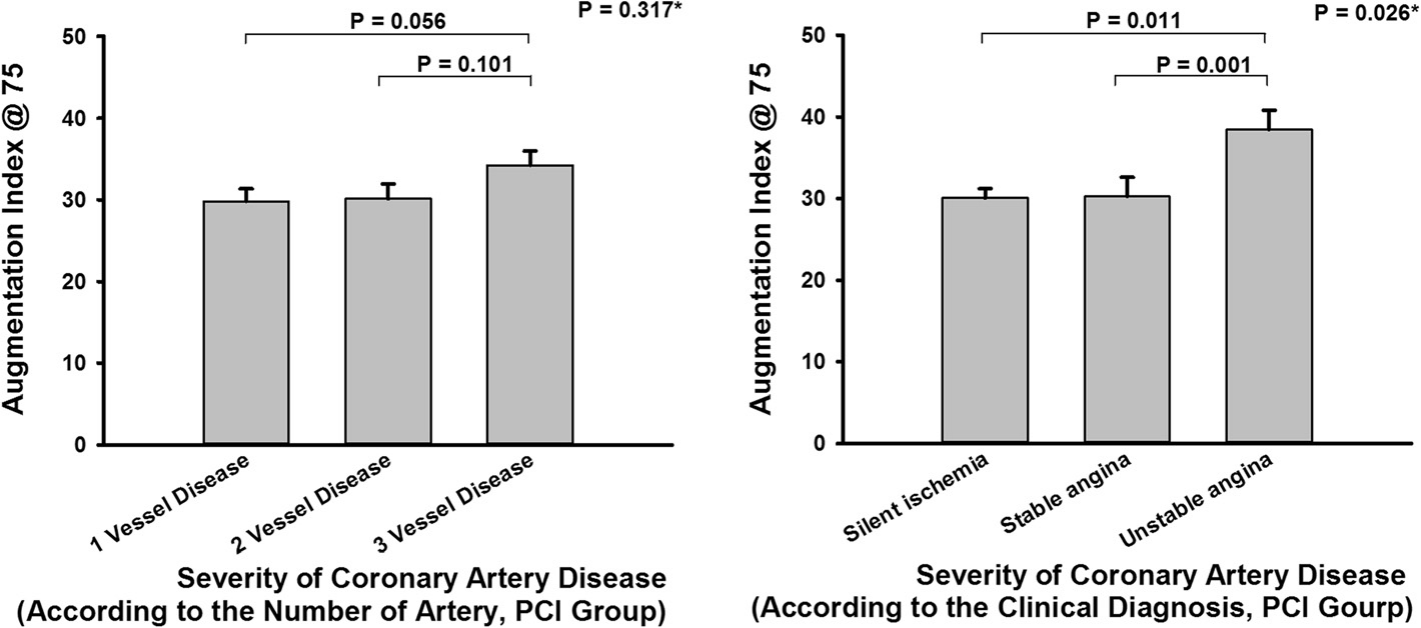
Augmentation index @ 75 and the severity of coronary artery disease. *ANCOVA test, adjusting for age, sex, ejection fraction. PCI, percutaneous coronary intervention

The AP (20.3 ± 9.5 mmHg), AIx (36.5 ± 9.3 %), and AIx@75 (31.6 ± 8.5 %) were higher in the PCI group than in the non-PCI group (16.9 ± 8.8 mmHg, 32.5 ± 10.0 %, 27.2 ± 9.0 %, respectively; Table 1). The AIx@75, adjusted for the left ventricular ejection fraction, was also significantly different between the PCI and the non-PCI groups (*n* = 111, p = 0.006). However, there were no significant differences between the PCI and the non-PCI groups with respect to brachial or central PP. The AIx@75 was significantly different for each clinical diagnosis in the PCI group (stable angina, 30.6 ± 7.7 %; silent ischemia, 30.2 ± 8.6 %; unstable angina, 38.5 ± 8.5 %; *p* = 0.035, adjusted for left ventricular ejection fraction, age, sex). The AIx@75 was consistently different among the disease severity classifications within the PCI group (1-VD, 29.8 ± 7.8 %; 2-VD, 30.1 ± 8.0 %; 3-VD, 34.3 ± 8.5 %; *p* = 0.109), but the differences were statistically insignificant (Fig. 1).

Statistically significant correlations between the risk factors based on the Framingham Heart Study and AIx@75 were observed in the non-PCI group (age, ρ = 0.383, p < 0.001; brachial PP, ρ = 0.223, *p* = 0.005; brachial SBP, ρ = 0.241, *p* = 0.002; height, ρ = −0.359, *p* < 0.001 and body mass index (BMI), ρ = −0.178, p = 0.025). Statistically significant correlations between the risk factors from the Framingham Heart Study and AIx@75 were observed in the PCI group (age, ρ = 0.473, *p* < 0.001; brachial PP, ρ = 0.378, *p* < 0.001; brachial SBP, ρ = 0.261, p = 0.026 and height, ρ = −0.514, *p* < 0.001). The AIx@75 was associated with age, sex, and brachial DBP, as determined using multiple linear regression analysis, in the non-PCI group. The AIx@75 was associated with age, sex, and smoking status using multiple linear regression analysis in the PCI group (Table 2).

**Table 2:** Correlation of augmentation index@75 with risk factors

	Non-PCI group				PCI group			
			Multiple regression				Multiple regression	
	ρ	p	r^2^	p	ρ	p	r^2^	p
			0.396	<0.001			0.488	<0.001
			β	p			β	p
Age	0.383	<0.001	0.250	<0.001	0.473	<0.001	0.282	0.014
Male			−4.333	0.029			−5.691	0.043
BPP (mmHg)	0.223	0.005	0.156	0.346	0.378	<0.001	0.071	0.243
BSBP (mmHg)	0.241	0.002	−0.145	0.323	0.261	0.026	0.106	0.263
BDBP (mmHg)	0.149	0.061	0.387	0.009	−0.079	0.507	0.006	0.796
Total cholesterol (mg/dL)	−0.026	0.748	0.005	0.718	0.067	0.578	0.039	0.648
HDL cholesterol (mg/dL)	0.150	0.071	0.03	0.608	−0.077	0.528	−0.310	0.057
Height (m)	−0.359	<0.001	−0.17	0.141	−0.514	<0.001	−0.193	0.470
BMI (kg/m^2^)	−0.178	0.025	−0.429	0.056	−0.081	0.497	−0.938	0.692
Hypertension			2.61	0.089			3.538	0.097
Diabetes			−3.161	0.067			0.043	0.983
Smoking			−2.294	0.146			6.142	0.007

*PCI* percutaneous coronary intervention, *BPP* brachial pulse pressure, *BSBP* brachial systolic blood pressure, *BDPB* brachial diastolic blood pressure, *HDL* high-density lipoprotein, *PCI* percutaneous coronary intervention, *BMI* body mass index

*p* < 0.05 considered significant; ρ, Pearson correlation analysis

### Analysis of correlation between VH-IVUS and AIx@75

**Fig. 2:**
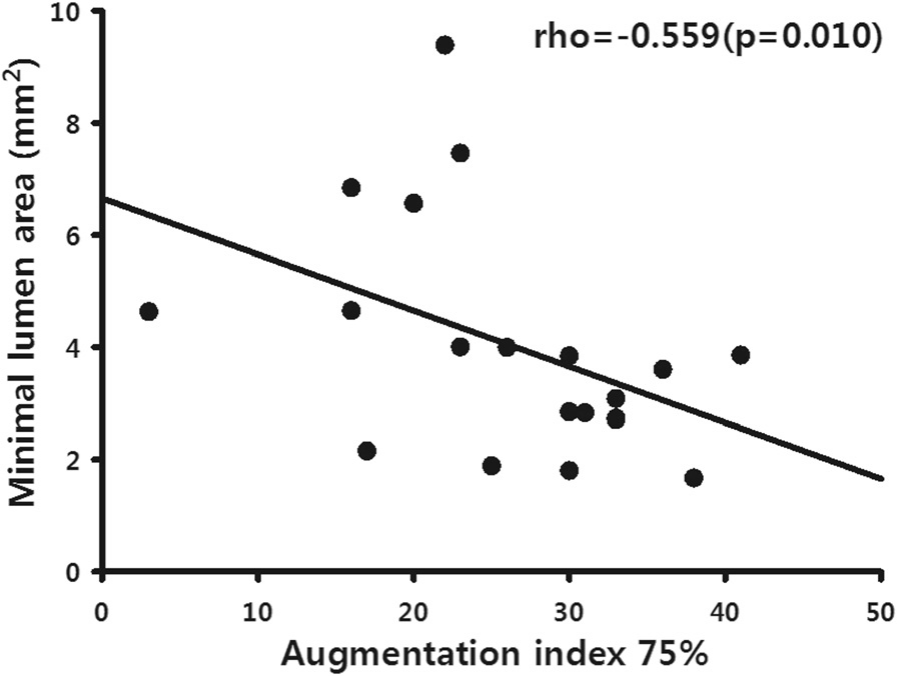
Relationship between minimal lumen area (determined using intravascular ultrasound) and the augmentation index @ 75

VH-IVUS was performed in 20 patients, revealing a mean MLA of 4.0 ± 2.1 mm^2^ and a mean dense calcium volume value of 10.7 ± 15.5 mm^3^. A significant, inverse correlation between the MLA and AIx@75 was observed (rho = −0.559, *p* = 0.010). The dense calcium volume and AIx@75 did not seem to be correlated (Table 3, Fig. 2).

**Table 3:** Analysis of the correlation between the virtual histology intravascular ultrasound parameters and the augmentation index @ 75 at the lesion site

	Augmentation index @ 75	
	rho*	p
EEM area (mm^2^)	−0.288	0.219
Minimal luminal area (mm^2^)	−0.559	0.010
Plaque and medial area (mm^2^)	−0.156	0.511
Fibrous volume (mm^3^)	0.371	0.107
Fibro-fatty volume (mm^3^)	0.141	0.553
Necrotic-core volume (mm^3^)	0.283	0.226
Dense calcium volume (mm^3^)	0.437	0.054
Fibrous volume composition (%)	−0.046	0.847
Fibro-fatty volume composition (%)	−0.163	0.493
Necrotic-core volume composition (%)	0.107	0.655
Dense calcium volume composition (%)	0.418	0.067

EEM, external elastic membrane

*p* < 0.05 considered significant; *rho, Spearman correlation analysis

### Cardiovascular risk points and AIx@75

A statistically significant correlation between the Framingham risk score and the AIx@75 was observed (ρ = 0.358, *p* < 0.001, *n* = 216). In a multivariate logistic regression analysis, the higher HDL (Odds ratio [OR], 0.963; 95 % confidence interval [CI], 0.937–0.989; *p* = 0.006), medication of hypertension (OR, 0.404; 95 % CI, 0.204-0.800; p = 0.009), and higher of BMI (OR, 0.891; 95 % CI, 0.805-0.985; *p* = 0.025) were associated with non-PCI. Higher of AIx@75 (OR, 1.048; 95 % CI, 1.009–1.088; *p* = 0.015) was independently associated with PCI (Table 4). The probability of PCI was calculated using a multivariate logistic regression analysis. The statistics related to the calculated probability of PCI were sensitivity, 31.4 %; specificity, 87.0 %; positive predictive value, 53.7 %; negative predictive value, 72.6 %; OR (the probability that the PCI group is related to the risk factors by the multivariate logistic regression analysis, compared to the non-PCI group), 3.06; relative risk (the probability that the risk factors by the multivariate logistic regression analysis are related to the PCI group compared to non-risk factors by the multivariate logistic regression analysis) 1.96. In ROC curve analysis of multivariate logistic regression model, higher HDL, medication of hypertension, and higher body mass index were associated with non-PCI and higher AIx@75 was associated with PCI (area under the curve, 0.764; 95 % CI: 0.701 to 0.819, z = 8.005; p <0.001).

**Table 4:** Logistic regression of the Framingham risk factors and the augmentation index @ 75 for the prediction of percutaneous coronary intervention

Variable	B	p	Odds ratio	95 % CI	B	p	Odds ratio	95 % CI
Age	0.038	0.052	1.039	1.000-1.080				
Male	−0.345	0.420	0.708	0.306-1.638				
BSBP (mmHg)	−0.034	0.846	0.967	0.688-1.359				
BDBP (mmHg)	0.071	0.685	1.073	0.763-1.508				
BPP (mmHg)	0.024	0.892	1.024	0.727-1.442				
BMI (kg/m^2^)	−0.132	0.022	0.876	0.783-0.981	−0.116	0.025*	0.891	0.805-0.985
Total cholesterol (mg/dL)	0.004	0.27	1.004	0.997-1.010				
HDL (mg/dL)	−0.043	0.006	0.958	0.929-0.988	−0.038	0.006*	0.963	0.937-0.989
Hypertension	−0.753	0.055	0.471	0.218-1.016	−0.905	0.009*	0.404	0.204-0.800
Diabetes	−0.137	0.725	0.872	0.407-1.869				
Smoking	0.358	0.390	1.431	0.632-3.237				
Augmentation index @75 (%)	0.040	0.092	1.041	0.994-1.090	0.047	0.015*	1.048	1.009-1.088

*BSBP* brachial systolic blood pressure, *BDPB* brachial diastolic blood pressure, *BPP* brachial pulse pressure, *BMI* body mass index, *HDL* high-density lipoprotein, *CI* confidence interval

*p* < 0.05 considered significant

*Variable by logistic regression analysis with a forward (conditional) selection

## Discussion

This study analyzed (1) the relationship between AIx@75 and CAD clinical severity, (2) the relationship between AIx@75 and IVUS findings, and (3) the association of a high CVD risk profile, based on the Framingham risk score, with the AIx@75.

### CAD and PWA

The AIx@75 was found to vary depending on the patient’s clinical diagnosis. Patients with unstable angina were found to have especially high AIx@75 values. A significant inverse correlation was observed, in this study, between the MLA, determined using IVUS, and the AIx@75. According to the analysis of the relationship between pulse wave velocity (PWV) and VH-IVUS [13], patients with higher PWVs had smaller MLAs. The AIx@75 values tended to differ depending on disease severity (1-, 2-, and 3- VD) and to correlate with the dense calcium volume in the arterial plaque, but the relationship was not statistically significant. This suggests that the dense calcium volume may not be associated with the clinical disease severity. The relationship between cardiac ischemia and PWA can be explained as an increased AIx, resulting from an increased central SBP (increasing myocardial oxygen demand) and a decreased central DBP (disturbed coronary blood flow), contributing to an imbalance between oxygen demand and supply; these factors occur due to ischemia [14]. Unfortunately, this theory does not explain the relationship between high AIx and unstable angina. According to recent studies, patients with high AIx values demonstrate poor outcomes [15]. Unstable angina, a manifestation of acute coronary syndrome, can be dangerous and may recur without appropriate intervention, supporting our findings of patients with unstable angina having high AIx.

### PWA and Framingham risk score

Risk score systems were developed to predict CVD in patients with atherosclerosis. Therefore, higher risk scores correlate with more frequent atherosclerotic events. Thus, a correlation is expected between these risk score systems and the AIx@75, as proven in many studies [6, 8, 16, 17]. In this study, we confirmed the correlation between the Framingham risk score and the AIx@75. However, the Framingham risk score has been suggested to possibly overestimate the risk in the Chinese populations [10]. Thus, the risk estimation may be affected by other races and environments, as well. The study population in the present study involved Asians and rural inhabitants. The patients had high risk scores and chest pain, leading to their undergoing CAG. Hence, this study attempted to determine whether PWA predicted the need for PCI in Koreans living in rural areas and having high-risk scores.

In this study, the AIx@75 was associated with age, sex, and smoking history for patients undergoing PCI; in the non-PCI group, the AIx@75 was associated with age, sex, and brachial DBP. Age has been a known determinant of the AIx@75 [18]. In a previous study, PWA was suggested to be a more sensitive predictor of risk in younger patients [8]. But, a different study suggested that the predictive power of PWA might not be limited to younger patients [15]. The smoking history was not associated with AIx@75 in young general population [19]. In the present study, the enrolled patients were elderly and had high risk factors, but those did not limit the predictive value of AIx@75.

The higher HDL, medication of hypertension, and higher of BMI were associated with non-PCI and higher of AIx@75 was associated with PCI in the study population, based on multivariable (Framingham risk factors; age, sex, brachial BP, BMI, medications for hypertension and diabetes, smoking, total cholesterol level, and HDL-cholesterol level) analysis. This is unlikely due to the low predictiveness of other risk factors, but due to the high-risk scores and normal BMI in the study patients. Interestingly, the probability of PCI, calculated using a multivariate logistic regression model (AIx@75, age, BMI and HDL), had a high OR and relative risk. Additionally, the patients with unstable angina had the highest AIx@75 values after each clinical diagnosis, adjusted for suspicious confounding factors. Therefore, AIx@75 was associated with PCI and clinical severity in Korean patients living in rural areas and having high Framingham risk scores.

PWA by an invasive study presents technical problems because the selection of inflection points is not easy. In this study, SphygmoCor® was used for PWA after applanation of the left radial artery. The device finds an inflection point mathematically and mechanically, following an analysis of the relationship between the second zero crossing of the fourth derivative and the peak flow time, using the formula: y = 0.91 + 1.31x; R = 0.75, where x is the time to the second zero crosses the fourth derivative, and y is the peak flow time [20]. Therefore, the AIx and related parameters can be measured easily, efficiently, and safely using this method.

PWA is well known to be related to CAD and the Framingham risk factors. PWAs, especially the AIx@75 values, also vary depending on the CAD clinical severity. The AIx@75, determined using PWA, was associated with PCI in patients with high Framingham risk scores.

### Limitations

This study has some limitations. First, this was a hospital-based observational study and the AIx@75 may have been affected by confounding factors. Additionally, echocardiography and HDL were not performed in all patients. However, verification by multivariate logistic regression analysis and analysis of covariance minimized this limitation. The second limitation of our study is the relatively small number of patients. The third, history of smoking, hypertension, obesity and diabetes was poor assessment. However, this study was based on risk factors from Framingham study. Therefore, the study could not consider all of the risk factors in detail. The fourth, according to a recent study, significant coronary stenosis tended to be determined by fractional flow reserve rather than the MLA by IVUS [21]. However, this study did not include the fractional flow reserve. Hence, a well-designed larger cohort study is needed to confirm these results and expand upon this limitation.

## Conclusion

The AIx@75 seems to reflect the clinical severity of CAD in the studied patient population. The AIx@75, determined using PWA, was associated with PCI in patients with high Framingham risk scores.

## Abbreviations

AIX: Augmentation index
AIX@75: Augmentation index adjusted by heart rate
AP: Augment pressure
AUC: Area under curve
BMI: Body mass index
CAD: Coronary artery disease
CAG: Coronary angiography
CVD: Cardiovascular disease
DBP: Diastolic blood pressure
MLA: Minimal luminal area
OR: Odds ratio
PCI: Percutaneous coronary intervention
PP: Pulse pressure
PWA: Pulse wave analysis
SBP: Systolic blood pressure
VD: Vessel disease
VH-IVUS: Virtual histology intravascular ultrasound
